# Stapled Transanal Rectal Resection for the Surgical Treatment of Obstructed Defecation Syndrome Associated with Rectocele and Rectal Intussusception

**DOI:** 10.5402/2012/652345

**Published:** 2012-03-25

**Authors:** Hesham M. Hasan, Hani M. Hasan

**Affiliations:** ^1^Department of General Surgery, Ain Shams University, Cairo, Egypt; ^2^Department of Obstetrics and Gynecology, AL-Azhar University, Cairo, Egypt

## Abstract

Obstructed defecation syndrome (ODS) is one of the most widespread clinical problems which frequently affects middle-aged females. There is a new surgical technique called stapled transanal rectal resection (STARR) that makes it possible to remove the anorectal mucosa circumferential and reinforce the anterior anorectal junction wall with the use of a circular stapler. This surgical technique developed by Antonio Longo was proposed as an effective alternative for the treatment of ODS. In this study we present our preliminary results with the STARR operation for the treatment of ODS. For this purpose, 40 consecutive female patients with ODS due to rectal intussusception (RI) and/or rectocele (RE) were recruited in this prospective clinical study, from May 2008 to October 2010. No major operative or postoperative complications were recorded, and after 12-month follow-up, significant improvement in the ODS score system was observed, and the symptoms of constipation improved in 90% of patients; 20% of patients judged their final clinical outcome as excellent, 55% as good, and 15% as moderate, with only 10% having poor results. After analyzing our results we can conclude that STARR is an effective and safe procedure for the treatment of obstructed defecation syndrome due to rectal intussusception and/or rectocele and can be performed safely without major morbidity.

## 1. Introduction

Obstructed defecation syndrome (ODS) is a frequently occurring condition that usually affects middle-aged females. This disease can affect the quality of life of many patients as these patients are obliged to spend several hours a day in the toilet; other symptoms of this disease include feeling of incomplete evacuation, excessive straining during defecation, the need for digital vaginal or perineal assistance, and the use of enemas or suppositories to defecate [1]. The etiology of ODS may be functional disorders, secondary to a spastic pelvic floor syndrome, in which failure to relax or paradoxical contraction of the anal sphincters muscles can cause the symptoms of ODS or anatomical rectal anomalies as rectal intussusception (RI) and/or rectocele (RE) [2]. By using anal 3-dimensional ultrasonography (3-DAUS), Regadas et al. [3] demonstrated that the anal canal is asymmetrical and that the internal anal sphincter is shorter in women, it is formed distally in the anterior upper anal canal weakening the anorectal junction that is devoid of striated muscle or any other anatomic support structure [4]. Thus, herniation starts in the anterior upper anal canal and anorectal junction wall as demonstrated by echodefecography and anal 3-dimensional ultrasonography (3-DAUS) technique, suggesting that these patients have anorectocele rather than rectocele [5].

Conservative therapy considered the first line of treatment in patients with ODS as more than 30% of these patients showed an improvement with diet and biofeedback therapy; also this line of management can avoid unnecessary and potentially dangerous surgery. Surgery should be reserved for patients with structural abnormalities who fail to respond to conservative treatment [6]. Patients who do not respond to conservative treatment are usually multiparous females affected by a combination of intussusception and rectocele; in these patients the correction of rectocele with a vaginal or perineal levatorplasty is often ineffective [6, 7].

Stapled mucosectomy for treatment of rectal mucosa prolapse and hemorrhoids was initially described in 1997 [8], and many publications have mentioned satisfactory results [9–12]. Recently, a new technique named stapled transanal rectal resection (STARR) developed by Antonio Longo has been described to treat the anorectal dysfunction such as rectocele and rectal intussusceptions [13, 14]. STARR involves a double stapling technique with the use of a circular stapler to remove the anorectal mucosa circumferential and reinforce the anterior anorectal junction wall correcting the structural abnormalities associated with ODS. Many publications demonstrated the safety and efficacy of this procedure for the treatment of ODS and the published results reported symptomatic improvement among those patients [15, 16].

In this study we present our preliminary results with the STARR operation for the treatment of obstructive defecation syndrome due to RI and RE.

## 2. Patients and Methods

From May 2008 to October 2010, 40 consecutive female patients with ODS caused by RE and/or RI were recruited in this prospective clinical study, which was performed at AL-Jedaani Hospital and Ibn Sena Medical College, Jeddah, Saudi Arabia.

All patients gave their written informed consent before participating in this study.

### 2.1. Inclusion Criteria

Patients with symptoms of obstructed defecation due structural abnormalities (rectocele and/or rectal intussusceptions) that failed to respond to conservative measures in the form of diet therapy, laxatives, enemas, and/or physiotherapy for more than six months and at least a score of 12 on obstructed defecation syndrome score (ODS-S) (Table 1).

**Figure 1:**
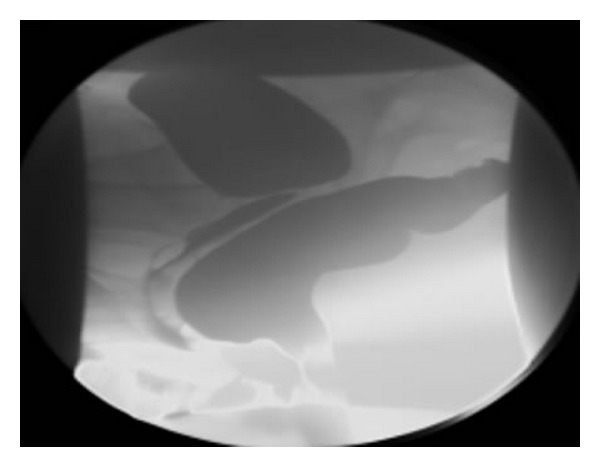
Cystocolpodefecography in sitting position during straining; the posterior colpocele is caused by a significant rectocele.

All the patients with an ODS-S ≥12 with RI (intussusceptions ≥10 mm) and/or RE (extending 2 cm or more from the rectal wall contour) shown by defecography (Figure 1). The presence of hemorrhoids was not a contraindication for inclusion in the study.

### 2.2. Exclusion Criteria

Patients with good response to conservative treatment, slow transit constipation, severe fecal incontinence, enterocele (grade 3, 4, and 5), and complete rectal prolapse of more than 3 cm and also patients with cystocele were excluded.

Preoperative clinical evaluation included complete history of presenting symptoms, numbers of pregnancies, history of episiotomy, and previous pelvic or anal surgeries. Clinical examination of the perineum, rectum, and vagina was performed to diagnose any associated diseases. Proctoscopy was performed for all patients to exclude any associated anorectal diseases.

**Figure 2:**
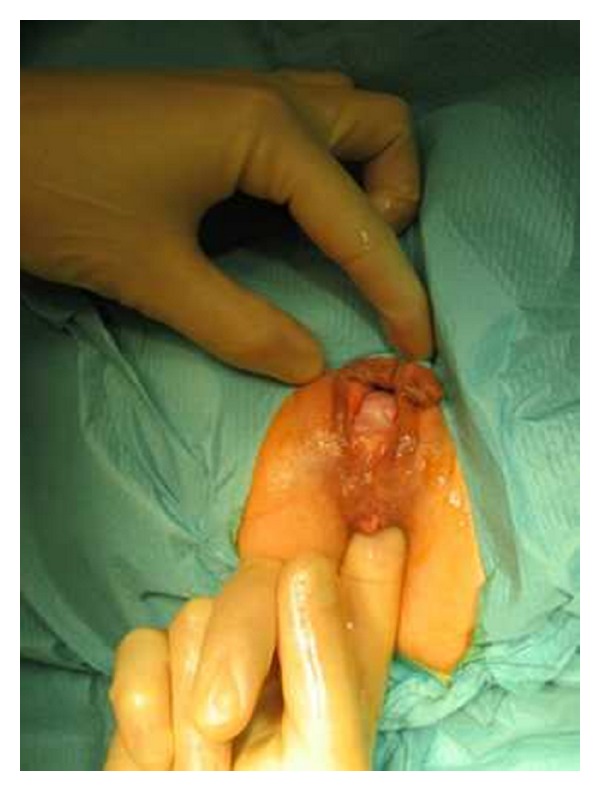
Anterior rectocele.

**Figure 3:**
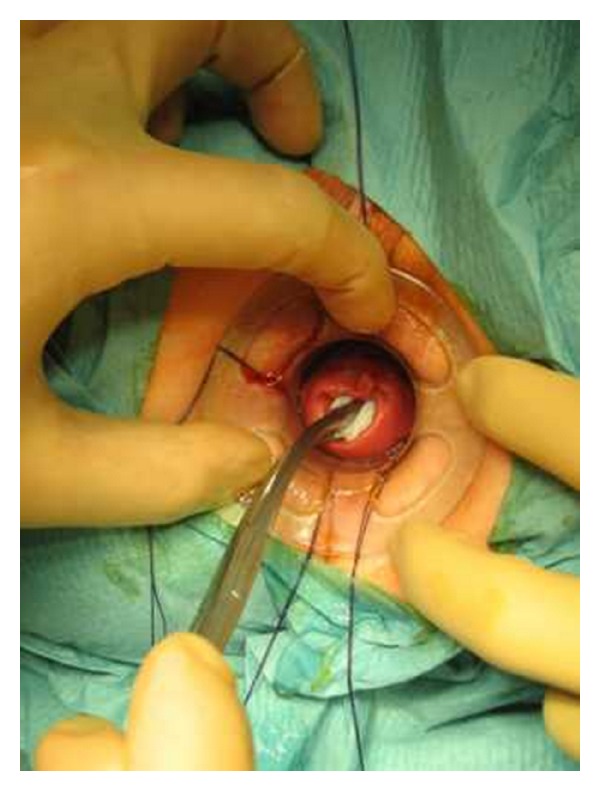
Apex of the rectocele was pulled down.

**Figure 4:**
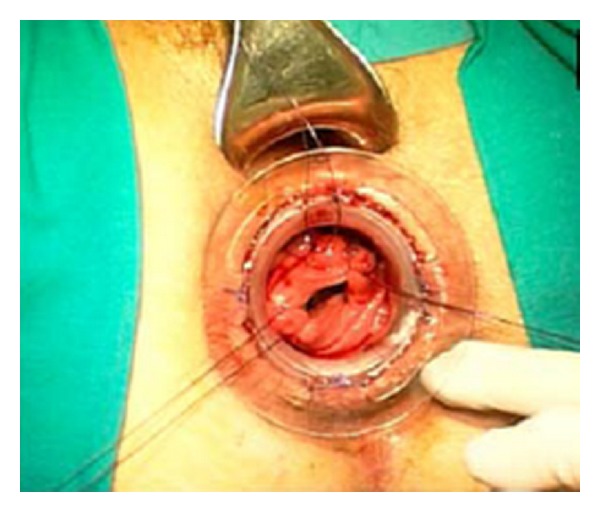
Three semi-circumferential. Purse-string sutures.

**Figure 5:**
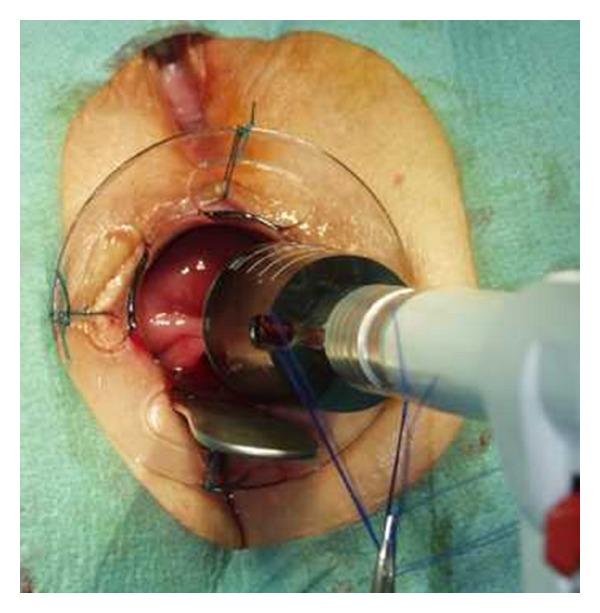
PPH-01 stapler.

 Preoperative preparation included one or two enemas at the morning of surgery, routine deep vein thrombosis prophylaxis, and perioperative broad spectrum antibiotics. General or spinal anaesthesia was used based on the individual anesthetist preference. Two circular PPH-01 staplers (Ethicon Endo-Surgery, Inc., USA) were used. The patient was placed in the lithotomy position. An initial examination was undertaken to confirm the presence and extent of the internal rectal prolapse and rectocele and also to confirm the absence of coexistent pathology (Figure 2). Circular anal dilator was inserted into the anal canal and maintained secured to the perianal skin with two stay sutures (anterior and posterior). The rectocele was pushed through the anal canal with a finger inserted into the vagina to identify its apex; the posterior vaginal wall was pulled up with a Babcock forceps, the apex of the rectocele was pulled down (Figure 3), and three semicircumferential purse-string sutures were positioned in the anterior rectum at approximately 1, 2, and 3 cm above the haemorrhoidal apex. The first PPH-01 stapler was inserted, and the posterior rectal wall was protected with a spatula. The ends of sutures were delivered through the specific holes of the stapler, and tension was applied to prolapse the removed tissues into the stapler housing, making sure that the posterior vaginal wall had not been incorporated; the stapler was closed and fired. By the same procedure, two semi-circumferential purse-string sutures and a second PPH-01 stapler were performed on the posterior rectal wall (Figures 4 and 5). Hemostatic stitches with full-thickness 2-0 Vicryl stitches were used to control bleeding from staples line. All surgical specimens obtained from procedure were sent for histological examination.

All patients had detailed data on preoperative status and perioperative and postoperative complications. A clinical assessment was performed at baseline and at 3, 6, and 12 months after surgery. The magnitude and degree of ODS were quantified by constipation scoring system (CSS) [17]. The validated CSS consists of five items, and the overall score ranges from 0 (normal) to 20 (severe constipation). The index of patient satisfaction was evaluated by a visual analog scale (VAS: with a score from 0 to 10), and a higher score suggests an improvement in patient satisfaction after the surgery.

### 2.3. Statistical Analysis

It was performed using paired *t*-test for continuous variables and Wilcoxon's signed-rank test for quantitative variables. A *P* value < 0.05 was considered statistically significant.

## 3. Results

During the period between May 2008 and October 2010 40 female patients with ODS caused by RE and/or RI (median age: 45.7 ± 12.3 years; range: 30–63 years) subjected to transanal rectal resection using PPH-01 staplers (Ethicon Endo-Surgery, Inc., USA) were included in this prospective study. All had been followed up for 12 months after surgery.

An anterior rectocele was present in 36 patients (90%), and 22 patients (55%) had an internal rectal prolapse and/or rectal mucosal prolapse. 32 patients (80%) had experienced 1–6 vaginal deliveries, 12 patients (30%) had experienced at least one episiotomy, and 18 patients (45%) had undergone prior anorectal or gynecologic surgeries. All patients had symptoms of obstructed defecation syndrome (Table 2).

The median operative time was  35 ± 10  minutes, and the median hospital stay was 1.7 ± 2.3 (ranging from 1 to 5) days; the specimen dimensions were 6.8 ± 2.5 × 9.7 ± 1.9 cm (height × width); rectal smooth muscle fibers were found in all the specimens. The only intraoperative complication was bleeding from the anastomotic ring, which occurred in 80% of cases and was secured with hemostatic stitches. The most common morbidity after surgery was defecatory urgency, and the incidence was 40% during the first postoperative week decrease to 10% after three months follow-up. Postoperative bleeding occurred in 4 (10%) patients, but it was minor and stopped spontaneously with conservative treatment with no further surgical intervention required. Other recorded complications were incontinence to flatus in 2 (5%) patients, acute urinary retention in 2 (5%) patients, persistent postoperative pain in 4 (10%) patients, and anal fissure in one (2.5%) patient. No staple line dehiscence, massive rectal hemorrhage, rectovaginal fistula, and perianal sepsis occurred, and also there were no postoperative mortality recorded (Table 3). At 12-month follow-up, the symptoms of constipation improved in 36 (90%) patients; however, constipation persisted or recurred in 4 patients after STARR procedure. There were a significant reduction in ODS scores at 12-month follow-up as compared with baseline (Tables 4 and 5).

Postoperative cinedefecography showed residual anorectoceles (grades I-II) in 6 (15%) patients and residual second-degree rectocele with internal mucosal prolapsed in 3 (7.5%) patients. As compared to preoperative defecographic findings, anterior rectocele was significantly reduced from 90% to 15% of patients (*P* < 0.001).

After 12-month follow up eight patients (20%) judged their final clinical outcome as excellent, 22 patients (55%) as good, 6 patients (15%) as moderate, with only four patients (10%) having poor results (Table 6).

## 4. Discussion 

ODS is a challenging clinical problem, the pathophysiology of which remains not clearly defined. RE and RI, however, are the two most frequent anatomic defects associated with ODS. Although various surgical procedures have been described for the treatment of the syndrome, many of these are unsuitable for patients accompanied with RE and RI [18]. Until the development of the STARR technique, there was no surgical procedure for correction of ODS, and patients were treated conservatively with diet and biofeedback therapy. In contrast to the transvaginal approach and perineal levatorplasty used to treat rectocele, the STARR procedure corrects both rectocele and rectal intussusceptions. Traditional operations in patients with both rectal mucosal prolapse and rectocele are associated with a high incidence of delayed healing of the perineal wound and dyspareunia. The combined endoanal and perineal approach increased the risk of sepsis due to fecal contamination and led to potentially fatal cases of pelvic gangrene [18]. 

STARR has been demonstrated as an alternative operation and a relatively noninvasive surgical technique for ODS caused by RE and RI. The novel procedure aims to correct rectocele, resect internal prolapse, restore anatomy, correct rectal volume, and improve function [19]. But it has been demonstrated that patient selection should be very careful because only symptomatic rectocele or rectal intussusceptions justifies surgical treatment; other associated pathologies such as irritable colon or pudendal neuropathy are not modified by operation, so symptoms may persist [20] A multicentric study done by Stuto et al. [21] demonstrated that STARR procedure, for management of ODS, is technically simple to perform and able to revert all constipation symptoms; the operative time and hospital stay were short, the postoperative pain and bleeding were minimal, there were no sepsis or postoperative dyspareunia, and patients return early to work. Several studies confirm the safety and efficacy of the STARR procedure for management of ODS [22–24]. Also, the data collected from this prospective clinical study suggest that more than 90% of our patients had a satisfactory surgical results with improved symptoms of ODS with the STARR procedure, coupled with a few intraoperative and postoperative complications. The only intraoperative incident was bleeding from the staple line, which occurred in 80% of patients, so the anastomotic ring should be meticulously checked and carefully secured with stitches whenever necessary, while the most common morbidity after surgery was defecatory urgency, and the incidence in our study was 40% during the first postoperative week decreasing to 10% after three-month follow-up. Other published studies have shown that defecatory urgency was the most common complaint in the immediate and intermediate recovery periods after STARR [24, 25]. Although the exact etiology of defecatory urgency is unclear, it may reflect the inflammatory response related to the staple line, presence of irritable rectum, and reduced rectal capacity or compliance. No major complications such as massive rectal hemorrhage and anastomotic line dehiscence occurred in our study. Few studies reported the incidence of severe complications such as staple line dehiscence, rectal diverticulum, pelvic infection, and even fulminating necrotizing pelvic fasciitis following the STARR procedure [26, 27]. Incontinence has been claimed to be a potential postoperative drawback of STARR; it may be a procedure-related complication caused by transient sphincteric impairment during instrumentation and anal dilatation [28–30]. In this study, only two (5%) patients complained of incontinence to flatus during the first two weeks after the procedures and improved within 3 months of surgery. Our results confirmed that the rate of postoperative pain was low and there were no cases of dyspareunia. Also, Edward et al. [31] in their prospective study concluded that STARR procedure is safe and effective, particularly in young females, due to the absence of complications related to the perineal levatorplasty and better results on postoperative pain, absence of dyspareunia, and better clinical outcome. Frascio et al. [32] in their trial on 30 patients reported no mortality or pelvic sepsis and 4% of postoperative bleeding treated surgically, while in our study postoperative bleeding occurred in 4 (10%) patients, but it was minor and stopped spontaneously with conservative treatment with no further surgical intervention required. 

 It is reasonable to suggest that the high percentage of successful results obtained, the short postoperative length of stay and the short time to return to work after the STARR procedure for management ODS would balance the relatively high cost of the procedure. 

## 5. Conclusion

Obstructed defecation syndrome (ODS) is one of the most widespread clinical problems that frequently affects middle-aged females. Rectocele (RE) and rectal intussusception (RI) are the two most common anatomic defects associated with ODS. STARR represents a true revolution in the surgical treatment of ODS caused by (RE) and/or (RI), and it appeared to be safe and effective with a successful outcome in most of the patients. Longer follow-up period, more than 12 months, may be needed to assess long-term functional outcomes and symptomatic recurrence.

## Figures and Tables

**Table 1:** Obstructed defecation syndrome score.

Symptoms	Never	Rarely	Sometimes	Usually	Always
Excessive straining	0	1	2	3	4
Incomplete rectal evacuation	0	1	2	3	4
Use of enemas/laxative	0	1	2	3	4
Vaginal/perineal digital pressure	0	1	2	3	4
Constipation	0	1	2	3	4

Never: 0 (never); rarely: <1/month; Sometimes: <1/week, ≥1/month;

Usually: <1/day, ≥1/week; Always: ≥1/day.

**Table 2:** Presenting symptoms.

Symptoms	Incidence
Excessive straining	32 (80%)
Feeling of incomplete evacuation	28 (70%)
Abdominal distension	22 (55%)
Feeling of rectal obstruction	14 (35%)
Rectal or vaginal digitation	12 (30%)
Laxatives more than 2 times/week	26 (65%)
Enema more than once/month	10 (25%)

**Table 3:** Postoperative complications.

Symptoms	First week	After 3 mo	After 6 mo	After 12 mo
Defecatory urgency	16 (40%)	4 (10%)	2 (5%)	1 (2.5%)
Postoperative bleeding	4 (10%)	0	0	0
Acute urinary retention	2 (5%)	0	0	0
Incontinence to flatus	2 (5%)	0	0	0
Pain	4 (10%)	2 (5%)	1 (2.5%)	0
Anal fissure	1 (2.5%)	1 (2.5%)	1 (2.5%)	1 (2.5%)

**Table 4:** Preoperative obstructed defecation syndrome score of the 40 patients.

Obstructed defecation score	No. of patients (%)
12–14	8 (20%)
15–17	26 (65%)
18–20	6 (15%)

**Table 5:** The obstructed defecation syndrome score before and at 12 months after the stapling procedure.

ODS symptoms	Preoperative, mean (SD)	12 months, mean (SD)	*t*-test	*P* value
Constipation	3.8 (2.04)	0.6 (0.42)	9.72	<0.001
Excessive straining	2.8 (0.92)	0.4 (0.32)	15.58	<0.001
Incomplete rectal evacuation	2.5 (1.06)	0.6 (0.86)	8.80	<0.001
Use of laxatives/enemas	3.3 (2.14)	0.7 (1.15)	6.77	<0.001
Vaginal/perineal digital pressure	1.8 (1.88)	0.0 (0.00)	6.06	<0.001
Total score	14.2 (9.13)	2.3 (2.9)	7.87	<0.001

*P < 0.001: highly significant.

**Table 6:** Subjective evaluation of outcome after surgery at six-month follow-up.

Subjective evaluation of outcome	No. of patients	%
Excellent	8	20%
Good	22	55%
Moderate	6	15%
Poor	4	10%

## References

[1] Kamm MA, Nicholls RJ, Dozois RR (1997). Constipation. *Surgery of the Colon & Rectum*.

[2] Keighley MRB, Keighley MRB, Williams NS (2000). Stipsi. *Chirurgia di Ano-Retto e Colon*.

[3] Regadas SMM, Regadas FSP, Rodrigues LV, Silva FR, Lima DMDR, Regadas-Filho FSP (2005). Importance of the tridimensional ultrasound in the anorectal evaluation. *Arquivos de Gastroenterologia*.

[4] Fritsch H, Hotzinger H (1995). Tomographical anatomy of the pelvis, visceral pelvic connective tissue, and its compartments. *Clinical Anatomy*.

[5] Pinheiro Regadas FS, Murad Regadas SM, Rodrigues LV (2005). New devices for stapled rectal mucosectomy: a multicenter experience. *Techniques in Coloproctology*.

[6] Singh K, Cortes E, Reid WMN (2003). Evaluation of the fascial technique for surgical repair of isolated posterior vaginal wall prolapse. *Obstetrics and Gynecology*.

[7] Watson SJ, Loder PB, Halligan S, Bartram CI, Kamm MA, Phillips RKS (1996). Transperineal repair of symptomatic rectocele with Marlex mesh: a clinical, physiological and radiologic assessment of treatment. *Journal of the American College of Surgeons*.

[8] Pescatori M, Favetta V, Dedola S, Orsini S (1997). Stapled transanal excision of rectal mucosa prolapses. *Tech Coloproctol*.

[9] Habr-Gama A, E Sousa AHS, Correia Roveló JM (2003). Stapled hemorrhoidectomy: initial experience of a Latin American group. *Journal of Gastrointestinal Surgery*.

[10] Nahas SC, Rodrigues Borba M, Teixeira Brochado MC, Sparapan Marques CF, Rizkallah Nahas CS, Miotto-Neto B (2003). Stapled hemorrhoidectomy for the treatment of hemorrhoids. *Arquivos de Gastroenterologia*.

[11] Sobrado CW, Cotti GCDC, Coelho FF, Da Rocha JRM (2006). Initial experience with stapled hemorrhoidopexy for treatment of hemorrhoids. *Arquivos de Gastroenterologia*.

[12] Wilson MS, Pope V, Doran HE, Fearn SJ, Brough WA (2002). Objective comparison of stapled anopexy and open hemorrhoidectomy: a randomized, controlled trial. *Diseases of the Colon & Rectum*.

[13] Longo A Treatment of hemorrhoidal disease by reduction of mucosa and hemorrhoidal prolapse with a circular suturing device: a new procedure.

[14] Altomare DF, Rinaldi M, Veglia A, Petrolino M, De Fazio M, Sallustio P (2002). Combined perineal and endorectal repair of rectocele by circular stapler: a novel surgical technique. *Diseases of the Colon & Rectum*.

[15] Ayav A, Bresler L, Brunaud L, Boissel P (2004). Long-term results of transanal repair of rectocele using linear stapler. *Diseases of the Colon & Rectum*.

[16] Renzi A, Izzo D, Di Sarno G, Izzo G, Di Martino N (2006). Stapled transanal rectal resection to treat obstructed defecation caused by rectal intussusception and rectocele. *International Journal of Colorectal Disease*.

[17] Agachan F, Chen T, Pfeifer J, Reissman P, Wexner SD (1996). A constipation scoring system to simplify evaluation and management of constipated patients. *Diseases of the Colon & Rectum*.

[18] Boccasanta P, Venturi M, Calabrò G (2001). Which surgical approach for rectocele? A multicentric report from Italian coloproctologists. *Techniques in Coloproctology*.

[19] Boccasanta P, Venturi M, Stuto A (2004). Stapled transanal rectal resection for outlet obstruction: a prospective, multicenter trial. *Diseases of the Colon & Rectum*.

[20] Van Dam JH, Hop WC, Schouten WR (2000). Analysis of patients with poor outcome of rectocele repair. *Diseases of the Colon & Rectum*.

[21] Stuto A, Boccasanta P, Venturi M (2000). Stapled transanal rectal resection (STARR) for obstructed defecation. A prospective multicentric trial. Annual Meeting Abstracts of American Society of Colon and Rectal Surgeons. *Diseases of the Colon & Rectum*.

[22] Reboa G, Gipponi M, Logorio M, Marino P, Lantieri F (2009). The impact of stapled transanal rectal resection on anorectal function in patients with obstructed defecation syndrome. *Diseases of the Colon & Rectum*.

[23] Arroyo A, González-Argenté FX, García-Domingo M (2008). Prospective multicentre clinical trial of stapled transanal rectal resection for obstructive defaecation syndrome. *British Journal of Surgery*.

[24] Jayne DG, Schwandner O, Stuto A (2009). Stapled transanal rectal resection for obstructed defecation syndrome: one-year results of the european STARR registry. *Diseases of the Colon & Rectum*.

[25] Titu LV, Riyad K, Carter H, Dixon AR (2009). Stapled transanal rectal resection for obstructed defecation: a cautionary tale. *Diseases of the Colon & Rectum*.

[26] Dodi G, Pietroletti R, Milito G, Binda G, Pescatori M (2003). Bleeding, incontinence, pain and constipation after STARR transanal double stapling rectotomy for obstructed defecation. *Techniques in Coloproctology*.

[27] Pescatori M, Zbar AP (2009). Reinterventions after complicated or failed STARR procedure. *International Journal of Colorectal Disease*.

[28] Gagliardi G, Pescatori M, Altomare DF (2008). Results, outcome predictors, and complications after stapled transanal rectal resection for obstructed defecation. *Diseases of the Colon & Rectum*.

[29] Binda GA, Pescatori M, Romano G (2005). The dark side of double-stapled transanal rectal resection. *Diseases of the Colon & Rectum*.

[30] Corman ML, Carriero A, Hager T (2006). Consensus conference on the stapled transanal rectal resection (STARR) for disordered defaecation. *Colorectal Disease*.

[31] Ram E, Alper D, Atar E, Tsitman I, Dreznik Z (2010). Stapled transanal rectal resection: a new surgical treatment for obstructed defecation syndrome. *Israel Medical Association Journal*.

[32] Frascio M, Stabilini C, Ricci B (2008). Stapled transanal rectal resection for outlet obstruction syndrome: results and follow-up. *World Journal of Surgery*.

